# The impact of out-of-home care on brain development: a brief review of the neuroscientific evidence informing our understanding of children’s attachment outcomes

**DOI:** 10.3389/fnbeh.2024.1332898

**Published:** 2024-02-22

**Authors:** Paula S. Oliveira

**Affiliations:** Anna Freud, and Department of Clinical, Educational and Health Psychology, University College London, London, United Kingdom

**Keywords:** brain development, looked after children/children in care, attachment, reactive attachment disorder (RAD), disinhibited social engagement disorder, institutionalization, foster care

## Abstract

Researchers interested in the effects of early experiences of caregiving adversity have employed neuroscientific methods to illuminate whether and how such environmental input impacts on brain development, and whether and how such impacts underpin poor socioemotional outcomes in this population. Evidence is compelling in documenting negative effects on the individual’s neurodevelopment following exposure to adverse or disadvantaged environments such as institutionalization or maltreatment. Neuroimaging research focused specifically on attachment-relevant processing of socioemotional stimuli and attachment outcomes among children looked-after is scarcer, but largely consistent. This review begins by summarizing the key general brain structural and functional alterations associated with caregiving deprivation. Then, neuroscientific evidence that is more directly relevant for understanding these children’s attachment outcomes, both by employing social stimuli and by correlating children’s neural markers with their attachment profiles, is reviewed. Brief interpretations of findings are suggested, and key limitations and gaps in the literature identified.

## Introduction

Literature on the neurodevelopment of children currently or previously looked-after aims to understand whether and how such a significant experience in early caregiving is associated with measurable effects on the brain. This typically includes children looked-after in institutions (or orphanages) and foster care. Beyond the experience of disruption in their care and separation from previous caregivers, children looked-after have frequently been exposed to other adverse experiences which led to the removal from the care of their families in the first place, such as abuse and neglect. Being looked-after is consistently associated with poorer developmental outcomes in the socioemotional domain, including attachment behavior (e.g., Bakermans-Kranenburg et al., 2011).

Evidence compellingly shows that early exposure to adverse or disadvantaged rearing environments, including institutionalization and maltreatment, is associated with negative effects on the individual’s neurodevelopment (e.g., Fox et al., 2010). A persuasive assumption to explain this link is that failure of neglectful caregiving settings to provide many elements of an “expectable” rearing environment—such as access to a consistent caregiver and appropriate cognitive and emotional stimulation—can lead to neural alterations, possibly in the process of specification of neural circuitry and the pruning of neurons and synapses as well as alterations in other neurobiological systems (e.g., neuroendocrine) that are dependent on experience (Greenough et al., 1987; Nelson, 2007; Nelson et al., 2011; Pechtel and Pizzagalli, 2011).

Understanding the precise neural changes associated with exposure to these forms of environmental deprivation is a critical scientific goal, not only for improving our understanding of brain development, but also in advancing our capacity to effectively prevent and treat their psychological sequelae. Of these negative effects following adverse early caregiving experiences, difficulties in the socioemotional domain seem to be particularly persistent even after children are placed in more positive environments (e.g., Hodges and Tizard, 1989; Rutter et al., 2007; Kreppner et al., 2010).

This review aims to briefly synthesize what neural changes have been associated to growing up in out-of-home care and the contribution of such developmental differences to explaining these children’s poorer attachment outcomes. Yet, neuroimaging work specifically focused on attachment in children looked-after is scarce. Accordingly, it will be important to first review general effects of being looked-after on brain development, as well as neural correlates of attachment-relevant socioemotional processing, to contextualize the findings from the fewer studies measuring attachment outcomes in this population.

### Brain development and general effects

Neuroscientific studies with children placed in out of home care have documented differences in brain structure (i.e., anatomy) as well as function, when compared to peers continuously raised by their families.

Studies of brain anatomy, using magnetic resonance imaging (MRI), show that institutionally reared children have smaller volumes of both white and gray matter (Mehta et al., 2009; Sheridan et al., 2012), and altered structural integrity of white matter (Bick et al., 2015), when compared to home-reared counterparts. One of these studies is from the Bucharest Early Intervention Project (BEIP) with children living in Romanian institutions (which at the time were especially impoverished) who were randomly assigned, between 7 and 33 months of age, to remain at the institution or move to high-quality foster homes. While both groups with institutionalization experience, i.e., children who remained institutionalized and those who got moved to foster care, showed a similar reduction in gray matter volume, those who moved to foster placements showed an encouraging recovery in white matter volume and integrity (Sheridan et al., 2012). Parallels can be established with research assessing children exposed to maltreatment (compared to non-maltreated), where a similar reduction in cortical gray and white matter volumes has been reported, with important differences remaining even after controlling for their overall smaller brain size (De Bellis et al., 1999, 2002).

Despite these findings of overall differences in cortical volume, such differences do not seem to be distributed evenly across the brain, nor consistently so throughout development. Researchers have looked at specific regions of interest, particularly those known to be highly sensitive to the influence of early stress. Many studies have documented regional differences between children exposed to early caregiving adversity (mainly maltreatment, but also institutional rearing) and controls, with effects predominantly reported in the emotion regulation circuitry. This circuitry crucially involves areas of the prefrontal cortex (which has an executive role in higher-order functions such as cognitive control and emotional regulation) and the amygdala (a limbic structure key in emotion processing), as well as connectivity between them.

Differences have been found in both regions, among children looked-after. Specifically, reduced gray matter volume, blood flow and cortical thickness in some prefrontal areas, including the orbitofrontal cortex, have been reported in institutionally reared children (Chugani et al., 2001; McLaughlin et al., 2014). Similar reductions have been reported in children exposed to maltreatment (Hanson et al., 2010; De Brito et al., 2013; Fujisawa et al., 2019).

A great number of studies has focused on the amygdala, because it is critically involved in emotional processing and threat detection and is also very susceptible to the early environment. Yet, findings are not straightforward to interpret. While increased amygdala volume has been documented in samples who were maltreated or institutionally reared earlier in life, usually assessed during childhood, the opposite finding of reduced volume has been reported in studies with adolescents and adults (Mehta et al., 2009; Tottenham and Sheridan, 2009; Tottenham et al., 2010). This pattern of results suggests effects of timing of exposure and of assessment, whereby an initial stress-induced hypertrophy and hyperactivity of amygdala neurons eventually lead to neuronal atrophy or cell death by adulthood (Tottenham and Sheridan, 2009; Teicher and Samson, 2016). This account of amygdala development trajectory in the context of early caregiving adversity received support from a longitudinal study with post-institutionalized youth internationally adopted into the United States – where larger volumes were seen before 6.5 years of age, but smaller volumes from 11 onwards (VanTieghem et al., 2021). Beyond differences in volume, there are also indications of alterations in network connectivity involving the amygdala, among children who experienced early adversity. Notably, evidence suggests that type of adversity, namely abuse or neglect, is associated with alterations in the connectivity with different clusters of regions (Cheng et al., 2021).

Focusing now on white matter, differences have been documented in several fiber tracts among children exposed to early caregiving adversity. Looking at the corpus callosum as an example (given its crucial role as the largest white matter tract in the brain and its involvement in interhemispheric communication) evidence with children looked-after consistently shows a reduced volume and integrity of this structure (Sheridan et al., 2012; Bick et al., 2015). Similar findings have been documented in children who experienced maltreatment, including neglect (De Bellis et al., 1999, 2002; Teicher et al., 2004). As seen earlier regarding recovery in white matter more generally, findings from the BEIP suggest some capacity for recovery in corpus callosum volume for children randomized to high-quality foster care, when compared to the group of children who remained institutionalized (Sheridan et al., 2012).

Thus far I have summarized (s/f)MRI findings emerging from the literature with children looked-after. Remarkably consistent with such evidence is that obtained from electrophysiological research (i.e., using electroencephalography, or EEG, which records electrical activity along the scalp, and the related technique of event-related potentials, or ERPs, which are derived from time-locked EEG responses to discrete presentations of stimuli). Indeed, institutionally reared children have been found to show an overall reduced EEG power and reduced brain activation during EEG recordings.

The term ‘reduced power’ is used to describe the finding that institutionalized children show higher power in low-frequency (theta) and lower power in mid- to high-frequency (alpha and beta) bands, compared to never-institutionalized controls. This result was obtained in two different samples (Marshall et al., 2004; Vanderwert et al., 2010; Tarullo et al., 2011; Debnath et al., 2020), spanning a long period of development from the toddler years up until 16 years of age. This atypical concentration of power in lower frequencies has been interpreted to signify neural hypoactivation in children raised in institutions, which has been hypothesized to result from lack of stimulation from responsive caregivers during a sensitive period of development (Marshall et al., 2004; Tarullo et al., 2011). Findings from the BEIP provide further insight into this issue. Specifically, the association between institutional care and reduced EEG alpha-power in that sample could be partly explained by the mediating role of reduced cortical white matter volume in the children who remained institutionalized (Sheridan et al., 2012). Encouragingly, benefits from placing children in high-quality foster care before age 2 saw these children’s alpha power at age 8 to be comparable to that of never-institutionalized children. These findings suggest that institutional rearing has an impact in shaping brain anatomy in ways that alter neural activity, but also that intervening early by placing children in high-quality foster-care may be able to mitigate, to some extent, the deleterious effects of deprivation. Consistent with this result is the effect of an attachment-based intervention for families classified as at risk for maltreatment, where children in those families who received the intervention presented increased high frequency power years later (Bick et al., 2019).

In addition to the association between adverse early caregiving and an overall reduced EEG power, atypical patterns of hemispheric asymmetry have been documented in this population. Hemispheric asymmetry is a relevant index of brain function to test in this context, because there is literature associating right-hemisphere dominance with withdrawal behavior and negative emotionality (Davidson, 1992). The most compelling evidence from children exposed to caregiving adversity comes from the BEIP. In that sample, institutionalized children developed an atypical trajectory of hemispheric asymmetry, with a prolonged period of increased right hemisphere activation (until 42 months of age) and a blunted rebound in left frontal activation, meaning that by 8 years of age they had greater activity in the right than the left hemisphere, when compared to never-institutionalized children (and those who were placed in foster-care at earlier ages). Consistent with this result among the Romanian children, a right alpha power asymmetry has also been reported in two American samples, one of toddlers in foster care and the other of maltreated school-aged children living with their families (Curtis and Cicchetti, 2007; Blaisdell et al., 2020).

In summary, neuroimaging findings from children exposed to institutional rearing or maltreatment present a picture of reduced volume in stress-sensitive brain structures and reduced brain electrical activity, as well as an altered trajectory of hemispheric asymmetry, which seem to result from their negative early caregiving experiences. It has been hypothesized that these effects develop from the lack of expected environmental input which the nervous system requires for typical development to unfold. Nevertheless, evidence points to the crucial intervention potential of improving caregiving to alter children’s developmental trajectories. What these studies cannot tell us is whether such differences in brain structure and function translate to children’s social phenotypes, and why some children with these adverse experiences show attachment difficulties while others do not. To examine this issue, research on socioemotional processing and on attachment behavior correlates is required, and it is reviewed next.

### Neuroimaging during processing of socioemotional stimuli

Both fMRI and ERP methods have been used to explore whether there are differences in how children with experience of adverse early care process attachment-relevant social cues, specifically faces as highly salient and relevant stimuli.

fMRI work with this population has substantially focused on regions and circuits critically involved in two types of processing: reward and threat. In general, a reduced activation in circuits related to reward (Mehta et al., 2010; Goff et al., 2013) and increased for threat processing have been found. Here we focus on the latter, as the paradigm of choice has been to present participants with face stimuli.

Research on threat detection has found increased amygdala activation in response to emotional faces in children adopted from care (Maheu et al., 2010; Tottenham et al., 2011), in line with the more abundant evidence from individuals exposed to child maltreatment (McCrory et al., 2011; Jenness et al., 2021). Unsurprisingly, this increased amygdala activation is accompanied by decreased activation in regions supporting cognitive control (Tottenham et al., 2011; Jenness et al., 2021). Increased amygdala reactivity has been hypothesized to serve an adaptive function in maltreated individuals, in allowing enhanced capacity for detecting threatening stimuli (McLaughlin et al., 2016), even if it may lead to later difficulties, including psychopathology (Jenness et al., 2021).

ERP studies of children looked-after while they view faces have investigated processing of face familiarity and processing of emotional expressions. To investigate the former, researchers have presented to children pictures of their caregiver and pictures of a stranger while recording their neural responses. Consistently across different samples exposed to varying levels of caregiving deprivation (including the Romanian children from the BEIP, children in institutional care in Portugal, and children in foster care in Germany), looked-after toddlers and pre-schoolers showed reduced ERP amplitudes in components involved in face processing, when compared to home-reared controls (Parker et al., 2005a; Moulson et al., 2009b; Kungl et al., 2017; Oliveira et al., 2023). These components include the P1, which indexes early and low-level feature processing of visual stimuli, and the N170, which occurs after the P1 and is a marker of more elaborate face-sensitive perceptual processes. Nevertheless, all groups of children were able to discriminate, at the neural level, the caregiver from the stranger’s face, indicating a preservation of this ability even in those exposed to multiple rotating caregivers (Parker et al., 2005a; Moulson et al., 2009b; Oliveira et al., 2023).

Effects of variation in the level of caregiver deprivation or lack of individualized care were seen in both the BEIP and the Portuguese samples. Specifically, for children in the BEIP who were randomized to foster care following institutionalization, amplitudes in the P1 were intermediate between those who remained institutionalized and those who were continuously raised by their birth families, suggesting positive effects of the improvement in caregiving. In the Portuguese institutionalized group, smaller amplitudes in the P400 component were observed in those living in placements with a poorer ratio of children-to-caregivers (i.e., more children per adult), highlighting the importance of consistent and individualized caregiving.

Children from the BEIP also completed ERP tasks of discrimination and recognition of faces posing different emotional expressions (Parker et al., 2005b; Moulson et al., 2009a; Nelson et al., 2013). Those who were institutionalized showed differences in patterns of processing of facial emotion when they were infants/toddlers, and again at age eight, but not during the preschool years. In the preschool assessment the task required simple emotion discrimination, whereas at age eight the task involved emotion recognition and was more demanding, perhaps justifying the discrepant results. Nevertheless, it was not clear which emotions were more affected by institutional rearing, as in the early years the main differences were in regard to sad and fearful emotions, while at age eight they were seen mostly for anger. Lastly, deficits in emotion recognition appeared to be remediable, given that subtle improvements in processing facial emotion were observed among the group moving from the institution to high-quality foster care.

In summary, blunted neural responses have been observed in children exposed to different levels of caregiver deprivation, with both institutional and foster care associated with some alterations in face familiarity processing. This indicates that the signs of reduced brain electrical activity described earlier apply specifically to the processing of stimuli that are highly relevant to attachment, that is the faces of caregivers (vs. other adults). The impact of care experience on the processing of emotional faces has been demonstrated in terms of increased amygdala activation to threatening stimuli, but electrophysiological responses to emotional faces requires further investigation.

### Neuroimaging and attachment phenotypes

Neuroimaging methods have the potential to contribute to our understanding of the increased rates of socioemotional and attachment difficulties observed in children who have been exposed to caregiving adversity. In this section we summarize neuroimaging findings on attachment security and the more extreme forms of attachment problems, namely those captured by the labels of Reactive Attachment Disorder (RAD) and Disinhibited Social Engagement Disorder (DSED) in children looked-after.

First focusing on attachment quality, two studies have linked neural correlates with attachment security among children in care. The first was another piece of work from the BEIP (Almas et al., 2012), which reported that alpha power at 8 years of age moderated the relation between children’s attachment security to the primary caregiver (at 42 months) and later social skills, among all children who experienced institutionalization. Specifically, only for children with higher alpha power, did a greater attachment security significantly predict better social skills. The second study, also cited already, assessed children in foster care in Germany (Kungl et al., 2017). In addition to the group differences we have seen for ERP amplitudes, researchers also found that variation in attachment security played a role in children’s face-familiarity processing, with insecurely attached children showing a reduced N170 component compared to secure ones. Even if preliminary due to the small sample size, these results may be interpreted as increased face expertise in secure children, beyond the experience of foster care, which we may speculate has been allowed by contingent social interactions within an adequately stimulating social environment provided by their caregiver (Kungl et al., 2017).

Another line of work has focused on more extreme forms of attachment difficulties which are intimately associated with experiences of early caregiving neglect, namely symptoms of RAD and of DSED.

A few different teams have employed neuroimaging tools to study this topic. An EEG study with post-institutionalized adopted toddlers found that their pattern of EEG power distribution (described earlier), namely a concentration in lower frequencies, predicted their display of socially disinhibited behavior later on at 36 months of age (Tarullo et al., 2011). Another team used fMRI with post-institutionalized adopted youth, who showed reduced differentiation in amygdala activation between the mother’s and a stranger’s face (compared to controls), which correlated with their disinhibited behavior; also, these effects were particularly prominent in children who were adopted later in life, suggesting effects of timing of removal from institutional care (Olsavsky et al., 2013). Consistent with results from both these studies were obtained from ERPs with a relatively large sample of Portuguese institutionalized pre-schoolers (Oliveira et al., 2023). Among these children who were currently living in institutional care, higher levels of DSED symptoms were associated with: (a) smaller P1 amplitudes (for both caregiver and stranger’s faces) and (b) smaller P400 differences in amplitude to each face, as well as a smaller P400 amplitude in response to the stranger’s face specifically—even after controlling for chronological and mental age.

In contrast to what was found for DSED, RAD symptoms did not predict any neural responses in the ERP components assessed in the Portuguese institutionalized pre-schoolers (Oliveira et al., 2023). However, a diffusion tensor imaging (DTI) study of previously maltreated youth with a RAD diagnosis living in a child welfare facility in Japan did show alterations to white matter microstructural integrity (Makita et al., 2020). Specifically, these youth showed alterations in the structure of the corpus callosum and pathways that are important for emotion regulation—alterations that, as we have seen, are associated with being looked-after more generally. The same team also reported reduced striatum activity in youth with RAD, during a monetary reward task (Takiguchi et al., 2015), therefore extending previous literature of the general impact of maltreatment and institutional rearing on the reward circuit.

To conclude, brain alterations observed in institutionally reared children, such as reduced EEG power and blunted ERP components, as well as alterations in white matter integrity, are associated with poor attachment outcomes, contributing to our understanding of difficulties that many children display following caregiving adversity. Such findings also provide preliminary insight into the underlying contributors explaining the heterogeneity in socioemotional functioning in this population, with a clear implication of the generic brain differences reviewed earlier for this specific phenotypic domain. Finally, the establishment of secure attachment relationships shows a preliminary buffering effect against the negative impact of adversity on these neural systems.

## Conclusion

This review provides a brief, and necessarily limited overview of the neuroimaging literature that is relevant for our understanding of the socioemotional development and, specifically, attachment outcomes in children looked-after. Evidence indicates widespread neural alterations in both brain structure and function among children who have experienced early caregiving adversity. Such alterations may be speculated to result from a protective adaptation to their adverse circumstances and/or damage to developing systems that are very sensitive to insults from the environment. These alterations are likely to partly underlie these children’s increased rates of socioemotional difficulties, including those captured by the DSED label, which tend to persist in some cases even after improvement in care (Zeanah et al., 2003). There are, nevertheless, encouraging examples of children’s capacity for recovery and resilience, and the positive impact of improved caregiving.

Our ability to generalize from these findings, particularly regarding longitudinal trajectories of brain structure and function, is limited by the fact that the studies included here span across different time points and developmental stages, and that many findings come from one single extreme setting (the BEIP). Another significant limitation of this work is the confounding overlap and lack of differentiation between different experiences of adversity. We know that often these children are exposed to different combinations of maltreatment, relationship disruptions and suboptimal caregiving, but most studies were not able to account for this.

Despite this limitation, all children in the studies included herein have been exposed to major adversity in the relationships with those that are supposed to nurture and protect them at a key period in their development. Indeed, the different types of caregiving adversity have in common well documented socioemotional and neurodevelopmental effects (Smith and Pollak, 2021). However, research linking early caregiving adversity, brain mediators and attachment outcomes is still scarce. While existing evidence supports the view that it is social neglect from being reared in unstable and unresponsive caregiving contexts what seems to be the key mechanism underlying both brain and attachment effects (beyond global deprivation or lack of cognitive stimulation; Tarullo et al., 2011; Bick et al., 2019; Blaisdell et al., 2020; Oliveira et al., 2023), more research is needed to verify this assumption, as well as to better understand the environment-brain-attachment links. Presently, any mechanistic interpretations are tentative.

Across development, and critically in the first few years of life, both experience-expectant and experience-dependent processes drive the growth and organization of the nervous system, with cascading effects of these dynamic processes on behavior (Greenough et al., 1987; Marshall and Kenney, 2009; Rutter, 2012). Environmental insults from neglectful or otherwise adverse caregiving experiences might contribute to alterations in neural pruning, axonal organization and myelination that explain the effects summarized in this review (e.g., Nelson, 2007; Pechtel and Pizzagalli, 2011; Bick et al., 2019). Specifically, it is plausible that lack of contingent, responsive stimulation from nurturing caregivers leads to over-pruning of cortical gray matter and reductions in white matter or myelination (e.g., Sheridan et al., 2012; Bick et al., 2015, 2019). In addition, such brain alterations are likely to have a complex impact on behavior, with effects on basic skills such as inhibitory control and attention regulation likely contributing to the resulting socioemotional phenotypes, including DSED (Tarullo et al., 2011).

However, clear differences also exist in the adverse outcomes associated with abuse or neglect, and important differences in effects from institutional or foster care rearing as well as variation in quality within these, beyond their social address (Soares et al., 2014; Cassiers et al., 2018). Considering these nuances, agglomeration of findings and direct comparisons between samples will always be tentative until further rigorous research is available. Notwithstanding such limitations, current understanding allows us to highlight the importance of contingent, responsive caregiving for children’s neurodevelopment, and the urgency of providing timely intervention to this vulnerable group.

## Author contributions

PO: Writing – original draft, Writing – review & editing.
